# Research priorities in pediatric parenteral nutrition: a consensus and perspective from ESPGHAN/ESPEN/ESPR/CSPEN

**DOI:** 10.1038/s41390-021-01670-9

**Published:** 2021-09-02

**Authors:** Mark J. Johnson, Alexandre Lapillonne, Jiri Bronsky, Magnus Domellof, Nicholas Embleton, Silvia Iacobelli, Frank Jochum, Koen Joosten, Sanja Kolacek, Walter A. Mihatsch, Sissel J. Moltu, John W. L. Puntis, Arieh Riskin, Raanan Shamir, Merit M. Tabbers, Johannes B. Van Goudoever, Miguel Saenz de Pipaon, on behalf of ESPGHAN/ESPEN/ESPR/CSPEN Working Group on Pediatric Parenteral Nutrition, Christian Braegger, Jiri Bronsky, Wei Cai, Cristina Campoy, Virgilio Carnielli, Dominique Darmaun, Tamas Decsi, Magnus Domellof, Nicholas Embleton, Mary Fewtrell, Natasa Fidler Mis, Axel Franz, Olivier Goulet, Corina Hartman, Susan Hill, Iva Hojsak, Silvia Iacobelli, Frank Jochum, Koen Joosten, Sanja Kolacek, Berthold Koletzko, Janusz Ksiazyk, Alexandre Lapillonne, Szimonetta Lohner, Dieter Mesotten, Krisztina Mihalyi, Walter A. Mihatsch, Francis Mimouni, Christian Mølgaard, Sissel J. Moltu, Antonia Nomayo, Charles Jean Picaud, Christine Prell, John W. L. Puntis, Arieh Riskin, Miguel Saenz De Pipaon, Thibault Senterre, Raanan Shamir, Venetia Simchowitz, Peter Szitanyi, Merit M. Tabbers, Dirk Vlasselaers, Chris H. B. Van Den Akker, Johannes B. Van Goudoever, Anne Van Kempen, Sascha Verbruggen, Jiang Wu, Weihui Yan

**Affiliations:** 1 Department of Neonatal Medicine, University Hospital Southampton NHS Trust, Southampton, UK; 2 National Institute for Health Research Biomedical Research Centre Southampton, University Hospital Southampton NHS Trust and University of Southampton, Southampton, UK; 3 Neonatal Intensive Care Unit, Necker-Enfants Malades Hospital, Paris University, Paris, France; 4 France and CNRC, Department of Pediatrics, Baylor College of Medicine, Houston, TX USA; 5 Department of Paediatrics, University Hospital Motol, Prague, Czech Republic; 6 Department of Clinical Sciences, Pediatrics, Umeå University, Umeå, Sweden; 7 Neonatal Unit, Newcastle University, Newcastle upon Tyne, UK; 8 Newcastle University, Newcastle Upon Tyne, UK; 9 Department of Neonatal Intensive Care, CHU La Réunion, Saint Pierre, France; 10 Evangelisches Waldkrankenhaus Spandau, Berlin, Germany; 11 Erasmus MC-Sophia Children’s Hospital, Rotterdam, The Netherlands; 12 Children’s Hospital, University of Zagreb School of Medicine, Zagreb, Croatia; 13 Department of Pediatrics Ulm University, Ulm, and Neu-Ulm University of Applies Sciences, Neu-Ulm, Germany; 14 Department of Neonatal Intensive Care, Oslo University Hospital, Oslo, Norway; 15 The General Infirmary at Leeds, Leeds, UK; 16 Bnai Zion Medical Center, Rappaport Faculty of Medicine, Technion, Haifa Israel; 17 Institute of Gastroenterology, Nutrition and Liver Diseases, Schneider Children’s Medical Center, Sackler Faculty of Medicine, Tel-Aviv University, Tel Aviv, Israel; 18 Amsterdam UMC, University of Amsterdam, Vrije Universiteit, Emma Children’s Hospital, Amsterdam, The Netherlands; 19 Department of Neonatology, La Paz University Hospital, Red De Salud Materno Infantil Y Desarrollo E Samid, Universidad Autonoma De Madrid, Madrid, Spain; 20 University Children’s Hospital, Zurich, Switzerland; 21 University Hospital Motol, Prague, Czech Republic; 22 Shanghai Jiao Tong University, Shanghai, China; 23 Department of Paediatrics, School of Medicine, University of Granada, Granada, Spain; 24 Polytechnic University of Marche, Ancona, Italy; 25 Universite De Nantes, Nantes, France; 26 Department of Pediatrics, University of Pecs, Pecs, Hungary; 27 UCL Great Ormond Street Institute of Child Health, London, UK; 28 University Medical Centre Ljubljana, Ljubljana, Slovenia; 29 University Children’s Hospital, Tuebingen, Germany; 30 University Sordonne-Paris-Cite; Paris-Descartes Medical School, Paris, France; 31 Schneider Children’s Medical Center of Israel, Petach Tikva, Israel; 32 Carmel Medical Center, Haifa, Israel; 33 Great Ormond Street Hospital for Children, NHS Foundation Trust and UCL Institute of Child Health, London, UK; 34 Children’s Hospital Zagreb, University of Zagreb School of Medicine, University of J. J. Strossmayer School of Medicine Osijek, Osijek, Croatia; 35 kLMU E Ludwig-Maximilians-Universitat Munich, Dr. Von Hauner Children’s Hospital, Munich, Germany; 36 Department of Pediatrics, Nutrition and Metabolic Diseases, The Children’s Memorial Health Institute, Warsaw, Poland; 37 Ku Leuven, Leuven, Belgium; 38 Department of Pediatrics, Division of Neonatology, The Wilf Children’s Hospital, The Shaare Zedek Medical Center, Jerusalem, Israel; 39 Tel Aviv University, Tel Aviv, Israel; 40 Department of Nutrition, Exercise and Sports, University of Copenhagen, and Paediatric Nutrition Unit, Rigshospitalet, Copenhagen, Denmark; 41 Oslo University Hospital, Oslo, Norway; 42 Ev. Waldkrankenhaus Spandau, Berlin, Germany; 43 Laboratoire Carmen, Claude Bernard University Lyon 1, Hopital Croix Rousse, Lyon, France; 44 CHU De Liege, CHR De La Citadelle, Universite De Liege, Liège, Belgium; 45 Great Ormond Street NHS Trust, London, UK; 46 General University Hospital, First Faculty Of Medicine, Charles University in Prague, Prague, Czech Republic; 47 Kliniekhoofd Intensive Care Medicine, UZ Leuven, Leuven, Belgium; 48 Emma Children’s Hospital, Amsterdam UMC, Amsterdam, The Netherlands; 49 OLVG, Amsterdam, The Netherlands; 50 Department of Pediatrics and Pediatric Surgery, Intensive Care, Erasmus Mc-Sophia Children’s Hospital, Rotterdam, The Netherlands; 51 Xin Hua Hospital, Shanghai, China; 52 Department of Gastroenterology and Nutrition, Xinhua Hospital, School of Medicine, Shanghai Jiao Tong University, Shanghai, China

## Abstract

**Abstract:**

Parenteral nutrition is used to treat children that cannot be fully fed by the enteral route. While the revised ESPGHAN/ESPEN/ESPR/CSPEN pediatric parenteral nutrition guidelines provide clear guidance on the use of parenteral nutrition in neonates, infants, and children based on current available evidence, they have helped to crystallize areas where research is lacking or more studies are needed in order to refine recommendations. This paper collates and discusses the research gaps identified by the authors of each section of the guidelines and considers each nutrient or group of nutrients in turn, together with aspects around delivery and organization. The 99 research priorities identified were then ranked in order of importance by clinicians and researchers working in the field using a survey methodology. The highest ranked priority was the need to understand the relationship between total energy intake, rapid catch-up growth, later metabolic function, and neurocognitive outcomes. Research into the optimal intakes of macronutrients needed in order to achieve optimal outcomes also featured prominently. Identifying research priorities in PN should enable research to be focussed on addressing key issues. Multicentre trials, better definition of exposure and outcome variables, and long-term metabolic and developmental follow-up will be key to achieving this.

**Impact:**

- The recent ESPGHAN/ESPEN/ESPR/CSPEN guidelines for pediatric parenteral nutrition provided updated guidance for providing parenteral nutrition to infants and children, including recommendations for practice.
- However, in several areas there was a lack of evidence to guide practice, or research questions that remained unanswered. This paper summarizes the key priorities for research in pediatric parenteral nutrition, and ranks them in order of importance according to expert opinion.

## Introduction

Parenteral nutrition (PN) is crucial to treat children that cannot be fully fed by oral or enteral route. The revised European guidelines on pediatric PN were published in 2018 by the European Society of Paediatric Gastroenterology, Hepatology and Nutrition (ESPGHAN), the European Society for Clinical Nutrition and Metabolism (ESPEN), the European Society of Paediatric Research (ESPR) together with the Chinese Society of Parenteral and Enteral Nutrition (CSPEN). These guidelines, updated from the previous version from 2005 (ref. ^1^), are a huge achievement, representing several decades of research and knowledge accumulation in pediatric PN, summarized into recommendations for practice based on the available evidence and consensus opinion to provide up-to-date evidence for health professionals working with infants, children, and adolescents receiving PN^2^.

While much progress has been made in the past two decades in terms of the evidence base, during the process of guideline development the multidisciplinary working party of professionals identified multiple gaps in knowledge where there was a need for additional research. Knowing where further studies are needed is important for improving neonatal and pediatric care.

Defining research priorities may serve as a common rallying point to focus resources and attract scientists and scholars into collaborative work around these research goals. In this context, the ESPR Section on Nutrition, Gastroenterology and Metabolism, on behalf of ESPEN/ESPEN/ESPR/CSPEN, aimed to summarize research priorities in the field of pediatric PN based on the knowledge gaps identified during the generation of the recent guidelines, and highlight those which seem most important and urgent.

## Methods

The revised ESPGHAN/ESPR/ESPEN/CSPEN pediatric PN guidelines consist of separate papers for energy, individual macro- and micronutrient needs, fluid requirements, venous access, organizational aspects, home PN, standardized vs. individualized PN, and safety considerations for prevention and management of complications. For each paper, the lead author or their delegate was asked to summarize current gaps in knowledge that became apparent while compiling the revised guidance, and by liaising with co-authors. Research agendas were proposed that would help address key issues on that topic, together with some brief, relevant background information and/or justification for the research priorities identified. Two authors (M.J. and M.S.P.) then collated all the responses into a list of all research priorities identified (see Supplementary file 1), which was circulated to all lead authors of the original guidance for comment before being finalized.

Next, given that the original 2018 PN guideline authors identified multiple research gaps, we aimed to rank them in order of importance. An electronic survey was then performed through the online tool Limesurvey v 3.2 (LimeSurvey GmbH Survey Services and Consulting, Hamburg, Germany) between April 2020 and May 2020. A unique link to the questionnaire was sent to the 47 members of the ESPGHAN/ESPEN/ESPR/CSPEN Working Group on Pediatric Parenteral Nutrition (who were all involved in drafting the guidelines) and to the 41 members of the ESPR section on Nutrition, Gastroenterology and Metabolism (11 of whom were involved in the drafting the guidelines, and were already included as part of the ESPGHAN/ESPEN/ESPR/CSPEN group). All participants were therefore healthcare professionals with extensive experience in pedaitric nutrition and/or in managing PN from a wide range of European countries, Israel, and China. Non-responders received a reminder email after 2 weeks. For each topic area, clinicians and researchers working in the field were asked to choose a minimum of one and a maximum of three of the research priorities for each topic, and research priorities for each topic were then ranked based on the number of votes. To highlight the main overall priorities, respondents were asked at the end of the survey to select five of their previous choices across the survey that they felt were the most important overall across all the topics. Again, these were ranked according to the total number of votes for each priority.

## Results

The initial process consultation process carried out with the original ESPGHAN/ESPEN/ESPR/CSPEN guideline authors identified a total of 99 research priorities, 10 of which focussed specifically on the preterm infant population. The complete list of research priorities can be seen in Supplementary File 1. The initial survey for ranking the priorities was sent to 77 clinicians and researchers working in the field. Eleven responded to say that they were no longer working in the field or had a conflict of interest, leaving 66 potential respondents. There were 55 full responses and 4 partial, making an overall response rate of 89.4%. Thirty-five (59%) of respondents were male and 24 (41%) female. The resulting top five ranked priorities for each topic area following voting are shown in Table 1 (energy and macronutrients), Table 2 (fluid, electrolytes, calcium, phosphate, magnesium and micronutrients), and Table 3 (venous access, complications, organizational aspects, home PN and ready to use and standardized formulations).

**Table 1:** Key research priorities for energy and macronutrients in pediatric parenteral nutrition.

Topic (number of respondents)	Priorities	Number of votes	Rank
Energy (59)	For all children and infants who receive parenteral nutrition, understand the relationship between total energy intake, rapid catch-up growth and long-term metabolic function and neurocognitive outcomes	40	1
	In critically ill children, define optimal energy intake for different phases of illness (acute, stable, and recovery) and the optimal route and doses of macro- and micronutrients	32	2
	In preterm infants, define optimal energy:protein ratio for growth and later long-term metabolic function and neurocognitive outcomes	30	3
	For all children and infants who receive parenteral nutrition, establishing more robust evidence and recommendations for optimal energy intakes during PN	20	4
	In critically ill children, defining the energy:protein ratio for optimal body composition and clinical outcomes	13	5
	In children with traumatic brain injury, septic shock, burns, and severe undernourishment, establish energy requirements in the different phases of disease	13	5
Amino acids (57)	For critically ill children who receive parenteral nutrition, understand the pathophysiological mechanisms underlying the harmful effect of administering proteins in the acute phase of critical illness (e.g. impaired autophagy)	30	1
	In preterm infants, undertaking studies (ideally RCTs) to investigate the impact of different PN amino acid intakes on growth during PN	28	2
	In preterm infants, define the optimal glucose and lipid intakes for maximizing protein accretion and growth at various parenteral amino acid intakes	25	3
	In critically ill term infants and older children, establishing better data to enable firm conclusions on the advisable lower and upper limits for protein intake, based on optimizing short- and long-term clinical outcomes	25	3
	For critically ill children, define the optimal dose and composition of amino acid mixture for optimal short- and long-term clinical outcomes	24	5
Lipids (56)	In preterm infants, determine the initial, optimal and/or maximal dose of lipid infusion and the ideal fatty acid (FA) composition needed to achieve the best long-term effects on morbidity, growth, and neurodevelopment	39	1
	For all children who receive parenteral nutrition, understand the effect of the type and dose of different intravenous lipid emulsions (ILEs) on the reversal of intestinal failure associated liver disease (IFALD)	28	2
	For all children and infants who receive parenteral nutrition, clearly define an upper limit of lipid intake during sepsis based on optimizing short- and long-term clinical outcomes	26	3
	In preterm infants, develop lipid emulsions containing both DHA and AA at significant balanced amounts, and test their effectiveness on improving short- and long-term clinical outcomes	25	4
	For all children and infants who receive parenteral nutrition, understanding of the impact of timing of commencement and dosage of PN lipid on outcomes	15	5
Carbohydrates (56)	For preterm infants, establish a robust definition of hyperglycemia, and compare effectiveness of management using insulin to a reduction carbohydrate for its impact on mortality, morbidity, growth, and long-term metabolic function and neurocognitive outcomes	41	1
	In critically ill neonates and children, characterize the relationship between excessive glucose intake and dyslipidemia	24	2
	In critically ill infants and children, understand the consequences of hypoglycemia on long-term outcomes	24	2
	For all children and infants who receive parenteral nutrition, establish the potential benefits of increased glucose intake whilst avoiding hyperglycemia	22	4
	For all children and infants who receive parenteral nutrition, investigate endogenous glucose production rates in order to inform recommended glucose intakes	17	5

*RCT* randomized controlled trial, *PN* parenteral nutrition, *NEC* necrotizing enterocolitis, *FA* fatty acids, *ILE* intravenous lipid emulsion, *AA* arachidonic acid, *DHA* docosahexaenoic acid, *IFALD* intestinal failure associated liver disease.

**Table 2:** Key research priorities for fluid, electrolytes, calcium, phosphate, magnesium and micronutrients in pediatric parenteral nutrition.

Topic (number of respondents)	Priorities	Number of votes	Rank
Fluids and electrolytes (56)	For neonates during the initial postnatal period, define optimal fluid and electrolyte requirements, including electrolyte to macronutrient ratios	35	1
	For neonates with varying gestational age and birth weight, determine the course of weight loss and gain after birth for optimal outcomes	28	2
	For all children and infants who receive parenteral nutrition, understanding the short-, medium-, and long-term clinical effects of the metabolic acidosis associated with parenteral nutrition	21	3
	In preterm infants, neonates, and children, undertake studies (ideally RCTs) to determine the short- and long-term effects of fluid therapy with sodium and chloride concentrations similar to that of plasma for “maintenance hydration”	19	4
	During the transition phase after birth in neonates, understand the relationship between the fluids and electrolytes received in utero, birth hydration status, and weight loss	15	5
Calcium, phosphate, and magnesium (56)	For all children and infants who receive parenteral nutrition, explore the optimum surrogate parameter for monitoring bone mineral accretion.	28	1
	For all children and infants who receive parenteral nutrition, develop bedside tools to individually monitor bone mineral (microcrystalline apatite) accretion and bone mineral status ((Ca+P)/body weight)	24	2
	In preterm infants, understand the metabolic changes that occur on refeeding, including include target values of calcium, phosphate, and magnesium needed to stabilize electrolyte balances and improve bone mineralization considering nutritional intake and gestational age	22	3
	For all children and infants who receive parenteral nutrition, generate more evidence to enable robust recommendations for phosphate requirements based on calcium deposition and protein accretion in the same way as can be done for adults.	18	4
	In newborn infants, understand the minimum bone mineral accretion to achieve within the first 2–4 weeks of life	17	5
Iron and trace elements (56)	In children of in different age groups and different stress status who receive PN, define normal ranges for biomarkers of iron and trace element status	36	1
	In infants and children on long-term PN, identify how minimal enteral nutrition may meet needs for trace elements including copper, chromium, manganese, molybdenum, and selenium.	32	2
	For children on PN in different populations (very preterm infants, children below 2 years of age, term infants with gastrointestinal failure or infants with IFALD, older children, etc.), determine the safety, efficacy, and stability of different iron compounds	22	3
	Assess the compatibility/stability of available iron and trace element compounds in various PN solutions	19	4
	For all children and infants who receive PN, evaluate trace element contamination in PN products, e.g. manganese and chromium.	9	5
Fat soluble vitamins (56)	In preterm infants, determine the vitamin D content for PN for optimal short- and long-term outcomes	28	1
	For all children and infants who receive PN, understand the health effects of additional vitamin D supplementation (in addition to its role in calcium and phosphate metabolism), such as prevention of immune-related and infectious diseases, cardiovascular disease, and cancer.	20	2
	In preterm and VLBW infants, establish what constitutes an adequate supply and plasma concentration of vitamin A with regard to optimal short- and long-term clinical outcomes	19	3
	In both infants receiving oral vitamin K supplementation and those whose mothers have taken medications that interfere with vitamin K metabolism, define optimal dose of parenteral vitamin K to ensure sufficiency	16	4
	For all children and infants who receive PN, compare the delivery of vitamin A in an intravenous emulsion compared with repeated intramuscular injection in terms of the benefits on vitamin A status, safety, and acceptability	15	5
	For all children and infants who receive PN, establish the upper and lower limits for the dose of vitamin E needed for optimal outcomes	15	5
Water soluble vitamins (56)	For all children and infants who receive PN, explore the precise requirement of B vitamins (thiamine, riboflavin, pyridoxine, cobalamin, niacin, pantothenic acid, biotin)	35	1
	In children and infants who receive PN, understand of the role of folic acid in the establishment of an individual’s DNA methylation profile during development, including its involvement in methylation profiles and in turn long-term health, during the life course	31	2
	In preterm infants, investigate the benefits of additional folic acid supplementation over and above current recommendations, as this is currently is controversial	21	3
	For preterm infants, term infants, and older children, define the vitamin C requirements for optimal short- and long-term clinical outcomes	19	4
	In children and infants who receive PN, develop clinical indicators for mild and moderate vitamin C deficiency	19	4

*RCT* randomized controlled trial, *VLBW* very low birth weight, *PN* parenteral nutrition, *VLBW* very low birth weight, *DNA* deoxyribonucleic acid, *IFALD* intestinal failure associated liver disease.

**Table 3:** Key research priorities for venous access, complications, organizational aspects, home parenteral nutrition and ready to use and standardized formulations in parenteral nutrition.

Topic (number of respondents)	Priorities	Number of votes	Rank
Venous access (56)	In children and infants who receive PN, explore the use of heparin or other anticoagulant agents to prevent CVC occlusion or thrombosis, including optimal doses and most effective mode of administration	26	1
	In newborns and small children, determine the best landmarks for safe CVC tip positioning	21	2
	For all children and infants who receive PN, assess the efficacy of ultrasound guidance in preventing complications	20	3
	For all children and infants who receive PN, establish the most cost-effective use of line locks in clinical practice to prevent occlusion and infection	19	4
	In children and infants who receive PN, evaluate the most reliable dressing methods for short-term catheters, and for a tunneled CVC	15	5
Complications (56)	For pediatric patients on long-term PN, establish a more complete understanding of the pathogenesis of IFALD	30	1
	For all children and infants who receive PN, investigate the role of antibiotic, antifungal, and ethanol locks in CVCs as an adjunct to systemic therapy or as an alternative to line removal	21	2
	In children who receive PN who have CRBSI, define the optimal duration of therapy for treatment with or without catheter removal	19	3
	Establishing more drug and PN brand specific data regarding the impact of medications on PN stability. This also applies to interactions with specific equipment and tubing and will vary depending on concentrations and flow rates	18	4
	For pediatric patients who receive long-term PN, further explore the use of ursodeoxycholic acid for the prevention of PN-related cholestasis, including data on liver disease and long-term outcomes	18	4
Organizational aspects (56)	For all children and infants who receive PN, evaluate (ideally using RCTs) the choice of enteral feed (e.g. elemental vs. polymeric vs. extensively hydrolyzed formula) and feeding method (e.g. continuous vs. intermittent/bolus feeding) while on PN in terms of their effect on tolerance and energy and nutrient balance	28	1
	For all children and infants who receive PN, develop a basic monitoring protocol considered essential to ensure safety of patients receiving long-term PN, together with follow-up and monitoring, together with a minimal data set	23	2
	In all children and infants who receive PN, assessing the benefits of cycling PN on short- and long-term outcomes, and the optimal regimens for doing this in different patient groups	22	3
	For all children and infants who receive PN, clearly describe the essential elements of a PN ordering process aimed at minimizing the risk of errors	21	4
	For all children and infants who receive PN, develop a non-invasive test for small intestine bacterial overgrowth	21	4
Home parenteral nutrition (56)	For all children and infants who receive home PN collecting long-term outcome data, perhaps as part of an international database	20	1
	Developing pediatric standard formulations for pediatric home PN, particularly those that could be kept at room temperature	18	2
	In children and infants who receive Home PN, carry out large multicentre audits of new treatments for weaning PN, e.g. use of GLP-2 in long-term patients at home, as this would help drive practice and the research agenda	16	3
	In children and infants who receive Home PN, explore better strategies to prevent IFALD and other hepatobiliary disease	16	3
	For children and infants who receive Home PN, establish a network for performing multicentre clinical trials of home PN	14	5
	Understanding of the impact of home PN on quality of life of children and their families	14	5
Ready to use and standardized formulations (55)	For all children and infants who receive PN further evaluate the impact of standardized PN compared to individually tailored PN, including costs and outcomes	38	1
	Developing enhanced informatics systems for the prescription, administration, and assessment of PN in pediatric patients	26	2
	For neonatal and pediatric patients who require PN, developing multi-chamber PN bags, and assessing these in a formal trial	22	3
	Investigating whether each center is able to manufacture a composition of PN for neonatal and pediatric patients requiring PN, that is compliant with the current new recommendations, with consideration of the case for a standardized European PN prescription	19	4

*CVC* central venous catheter, *PN* parenteral nutrition, *CRBSI* catheter-related bloodstream infection, *RCT* randomized controlled trial, *GLP-2* glucagon like peptide 2, *IFALD* intestinal failure associated liver disease.

### Energy

There are still considerable gaps in knowledge regarding how energy needs differ in both different phases of critical illness (acute, stable, recovery), chronic illness, and in infants born preterm (Table 1). Acute injury, infection, or a surgical insult induce a metabolic response that is proportional to the magnitude, nature, and duration of the injury. This response is characterized by a brief hypometabolic phase followed by a hypermetabolic phase, which is catabolic in nature. The duration of this catabolic response in most critically ill children is unknown but might be short. The impact of acute and chronic illness on energy requirements in children is not well understood^3^. Recent work has highlighted the uncertainties around optimal energy intakes in critically ill children accounting for the endogenous production of energy in the acute phase of disease, suggesting that more work is also needed to define these for term and preterm infants^4^. In addition, the optimal proportions of energy delivered by carbohydrate and lipid in PN, and the optimal ratio of energy to protein remains unclear, particularly for preterm infants.

### Amino acids

In preterm infants, defining optimal amino acid intake is challenging and more evidence is needed regarding the amino acid intake (and the relative amount of energy) needed for optimal metabolic and neurodevelopmental outcomes. No studies exist looking at protein/amino acid intake in relation to (functional) outcome in critically ill children, though several studies suggest that nitrogen balance is improved by higher amino acid intakes. However, a secondary analysis of the PEPaNIC study reported that a higher total daily amino acid intake during the first week of pediatric intensive care unit (PICU) stay was associated with an increased likelihood of acquiring new infections, and a lower likelihood of both earlier weaning from mechanical ventilation and early live PICU discharge^5^. While these findings need to be interpreted with caution in light of the issues associated with such secondary analyses, coupled with the heterogeneous nature of the PEPaNIC study population, intersite differences and the impact of over- and undernutrition on outcome, the optimal timing, dose, and composition of an amino acid mixture for critically ill children remains unclear. Since high doses appear harmful during the acute phase of critical illness, further research in this area is urgently needed (see Table 1).

### Lipids

Further well-designed and adequately powered studies are necessary to determine the optimal and/or maximal dose of lipid infusion and the long-term effects on morbidity, growth, and neurodevelopment. In preterm infants, there is some evidence that they have lower amounts of docosahexaenoic acid (DHA) and arachidonic acid (AA) postnatally, which has been associated with increased risk of morbidities such as retinopathy of prematurity (ROP) and adverse neurodevelopmental outcome^6^. New lipid emulsions specifically designed for preterm infants and containing higher amounts of DHA and AA should be developed and tested. The impact of different intravenous lipid emulsions (ILEs) on inflammatory states, infection risk, and pulmonary hypertension is not fully understood and more high-quality randomized controlled trials (RCTs) are needed.

Several observational studies have reported the efficacy of pure fish oil (FO) ILE as monotherapy in the treatment of IFALD in infants and children, though from studies to date it is unclear whether reversal of cholestasis was because of decreasing lipid intake or the effect of FO itself (including the high α-tocopherol load) or a combination of each. Definitive studies in this area, particularly in relation to both the safety and efficacy of the long-term use of FO, would be beneficial^7^.

### Carbohydrate

The relationship between hyperglycemia and lipid metabolism is not fully understood, particularly in preterm infants, and it is not clear why some preterm infants are less able to tolerate intravenous glucose while others can metabolize normal or even higher intakes without adverse effects^3^. Data to help determine whether insulin use or reduction in glucose intake, or a combination of both, leads to the best short- and long-term clinical outcomes in preterm infants are required (Table 1). Similarly, there are limited data on the efficiency with which glucose is utilized, together with endogenous glucose production rates, for infants and children^3^.

### Fluid and electrolytes

There are major gaps in knowledge regarding the pathophysiological mechanisms underlying fluid balance and distribution in critically ill children, particularly preterm and extremely low birth weight (ELBW, <1000 g) and very low birth weight (VLBW, <1500 g) infants^8^ (Table 2). The movement of water between physical compartments in the immediate postnatal period, is also not fully understood^9^.

The impact of fluid therapy during the transition phase in the first few days of life is not well described, and an understanding of the fluid shifts during this period is vital to define the “optimal weight loss” and consequent fluid and electrolyte intake strategy, particularly in small for gestational age and ELBW infants^10^. Further work determining the optimal electrolyte to macronutrient ratios in PN for sodium, potassium, phosphorous, and other electrolytes is also a priority. Clinical trials to establish optimal fluid and electrolyte intake during mixed (parenteral and enteral) nutrition are also warranted, and there is interest in studying the consequences on the ratio of intracellular fluid and extracellular fluid (ECF), and its composition under the current PN recommendations.

For children and infants beyond the neonatal period, the most significant change relates to the use of isotonic fluid as an intravenous fluid for “maintenance hydration” in sick children^11–13^, especially during the first 24 h^8^. Fluid distribution is different depending on the osmolarity of the solution being used, with hypotonic solutions distributed in the intracellular compartment and isotonic maintenance solutions mainly remaining in the extracellular compartment^14^. The latter may imply a lower fluid requirement than the volumes estimated by the traditionally used Holliday-Segar formula^8^. An understanding of this relationship between fluid distribution and fluid requirements is currently lacking.

### Calcium, phosphate, and magnesium

There is currently no practical way of monitoring bone mineral (microcrystalline apatite) accretion at the bedside, and no good surrogate marker for monitoring bone mineral accretion. Therefore, it is not currently clear what amounts and ratios of calcium, phosphate and magnesium should be provided to ensure optimal growth and bone mineralization in children, particularly in the context of preterm infants and critically ill children. The optimal phosphate intakes for both stability, and growth and bone mineralization in the context of refeeding syndrome and critical illness should also be more clearly defined by research evidence, as should the optimal plasma phosphate concentration (Table 2).

### Micronutrients

Micronutrients are an important component in any PN regimen in children^15^, though iron is not routinely added to PN due to stability issues. Different approaches for meeting iron requirements of children on long-term PN, including assessment of stability of iron in different PN solutions and safety of different doses and different iron compounds have not been well investigated (Table 2). Studies which define requirements as well as toxicity of iodine, copper, chromium, manganese, molybdenum, and selenium at different developmental stages in relation to health outcomes would help inform practice (Table 2).

Regarding vitamins, there has been a relative paucity of new research and data in the past 30 years, and optimal doses and infusion conditions in preterms, infants, and children have not been established (Table 2). Current recommendations for vitamins doses are largely based on expert opinion and there is a pressing need for more research to establish these^15, 16^.

### Venous access and complications

Some major clinically relevant gaps in current knowledge regarding choice of appropriate central venous catheter (CVC), selection of site, method of insertion, nursing care, handling and hygiene remain, and represent priorities for future research (Table 3). There is little evidence from clinical trials relating to optimal CVC tip position in infants and children in order to minimize risk of complications. Defining reliable radiological landmarks to enable safe positioning of the CVC tip in infants and children and determining the role of ultrasound guidance in order to prevent complications are key areas of clinical uncertainty.

Diagnosis and management of catheter related sepsis, including new methods and actions to implement measures to reduce infection risk, are also an area of growing research interest^17^. Studies comparing dressing methods are scarce and of poor quality, leaving uncertainties regarding the most appropriate dressing method (see Table 3).

Over 80% of venous thromboembolism in newborns and >40% in older children are associated with CVC. Establishing effective techniques for maintaining CVC patency and preventing thrombosis are the most clinically relevant knowledge gaps requiring urgent studies.

### Organizational aspects of parenteral nutrition

Cyclical PN (i.e. infusion for <24 h continuously) may have physiological and psychological advantages for infants and children, though current recommendations are largely based on small studies that assessed physiological changes when cyclical PN was introduced^18, 19^.

There is no standard biochemical monitoring regimen for PN, and a minimum data set for stable patients at different ages should be defined in order to avoid unnecessary and expensive repeat testing. New ways of defining and assessing specific complications, such as IFALD (including liver fibrosis), need to be developed^20, 21^ and should include a non-invasive technique for the measurement of hepatic venous pressure gradient^22^ and further refinement of transient elastography^23^.

Small intestinal bacterial overgrowth is thought to contribute to development of IFALD but is difficult to diagnose^24^. Polymerase chain reaction (PCR) techniques using bacterial 16S ribosomal RNA primers^25^ have potential to serve as a guide for antibacterial therapy. A simple test for bacterial overgrowth is required to inform the need for interventions and their effectiveness. Similarly, there are limited data with regard to optimum feed type (in the absence of breast milk) and mode of enteral nutrition (continuous vs. bolus) for infants on PN^26^.

### Home PN

There are still some gaps in knowledge and unanswered questions in relation to the impact of Home PN (HPN) in terms of quality of life, benefits, and costs (Table 3). There is little evidence regarding optimal weaning strategies, and the potential for standardized solutions suitable for HPN is relatively underexplored. There is emerging evidence that the use of standardized home PN solutions may be comparable to individualized solutions in terms of maintaining growth and serum biochemistry, though larger studies are needed to confirm this^27^. The use of strategies to reduce complications such as infection and IFALD have not been researched thoroughly in the context of HPN.

### Ready-to-use standardized vs. individualized pediatric PN preparations

Large multicentre RCTs, or at least observational cohort studies based on large databases comparing the outcomes of infants and children given individually tailored PN preparations to those given standardized “ready-to use” PN solutions, would help to establish evidence-based recommendations, and more importantly specifically define the patients and conditions where individually tailored or standardized ready-to-use PN preparations are advantageous (Table 3). The other area in need for on-going research as well as industrial development is the improvement and sophistication of ready-to-use all-in-one PN preparations, alongside improved computer-based software to aid their prescription.

### Overall priorities in pediatric PN

Of the 99 total research priorities identified by the original ESPGHAN/ESPEN/ESPR/CSPEN guideline authors, 74 received at least one vote. Six received 10 or more votes and so were considered more important than others (these top six are shown in Table 4). The most important research priority was to understand the relationship between total energy intake, rapid catch-up growth, later metabolic function and neurocognitive outcomes. Research into the optimal intakes of macronutrients needed in order to achieve optimal outcomes also featured prominently. For the remainder, there were multiple topics that received totals of 9, 8, 7 votes, meaning that these top six were clearly the highest priority.

**Table 4:** Overall highest research priorities in pediatric parenteral nutrition.

Research priority	Number of votes	Rank
For all children and infants who receive parenteral nutrition, understand the relationship between total energy intake, rapid catch-up growth and long-term metabolic function and neurocognitive outcomes	23	1
In critically ill children, define optimal energy intake for different phases of illness (acute, stable, and recovery) and the optimal route and doses of macro- and micronutrients	16	2
For preterm infants, establish a robust definition of hyperglycemia, and compare to effectiveness of management using insulin to a reduction carbohydrate for its impact on mortality, morbidity, growth, and long-term metabolic function and neurocognitive outcomes	14	3
In preterm infants, determine the initial, optimal, and/or maximal dose of lipid infusion and the ideal fatty acid composition needed to achieve the best long-term effects on morbidity, growth, and neurodevelopment	13	4
In preterm infants, define optimal energy:protein ratio for growth and later long-term metabolic function and neurocognitive outcomes	12	5
For critically ill children, define the optimal dose and composition of amino acid mixture for optimal short- and long-term clinical outcomes	10	6

## Discussion

This international expert consultation highlighted 99 research priorities in the field of pediatric PN. This consultation was intended to define the most urgent research studies needed to determine effective strategies in quality improvement and patient safety. To achieve this goal, we established the six most important research priorities from all topic areas based on each respondent’s top five priorities.

The most important research priority identified was gaining an understanding of the relationship between total energy intake, the potential adverse effects of rapid catch-up growth on later metabolic function and potential neurocognitive benefit for all age groups. Research into the range of intakes of macronutrients needed in order to achieve optimal outcomes also featured prominently, and in particular the ratio of energy to protein and the optimal dose and composition of amino acid mixture for optimal short- and long-term clinical outcomes. The latter is of interest as, despite the fact that inadequate protein balance and poor growth have been associated with currently available commercial mixtures of essential amino acids^28^ and conditionally essential amino acids^29^, there is little current research into modifying current commercial mixtures to try and improve outcomes, due to the complexity and expense this would involve. Of the 99 priorities identified, 15 of these related specifically to preterm infants, which means they are perhaps slightly overrepresented compared to other groups. Most of the research needs for preterm infants related to understanding basic nutrient requirements and their impact on long-term outcomes, particularly neurodevelopment.

An overarching theme across all nutrients, but macronutrients in particular, was that research into the intake required in order to achieve optimal outcomes was needed. This is a broad and challenging task. How to define and measure optimal outcomes remains a challenge in many areas of medicine, but especially so in pediatrics, given that long-term outcomes also involve optimal neurological, cognitive, and functional development and the impact of nutrition and growth in early life on the risk of non-communicable diseases such as coronary heart disease and metabolic syndrome in adulthood. Defining which outcomes are important to clinicians, patients, families, and other stakeholders is essential, and central to this is the work of the Core Outcome Measures in Effectiveness Trials (COMET) initiative^30^. Given the relatively small numbers of patients who receive PN, the use of registries and national databases is also needed to improve our understanding of practice and outcomes in this area. Such systems could also be used to drive larger trials using newer methodologies such as randomized point-of-care trials or registry trials as an alternative to conventional RCTs.

A limitation of the work described by this paper is that the authors and survey respondents came primarily from European countries, with some from China and Israel. The results of this work therefore reflect the priorities of those countries and are not a truly global international consensus. Furthermore, the guideline authorship group was drawn from the members of the contributing societies by volunteers and nominations, and so may reflect the priorities of those individuals to some extent, so may not be fully representative of the groups surveyed. In addition, families or patient groups drawn from those who require PN in infancy and childhood were not included. Furthermore, another potential limitation of the current approach to ranking is that it was a simple vote-based process. The intention of the paper was to collate all the research gaps that were identified as part of the process of researching and writing the 2018 ESPGHAN/ESPEN/ESPR/CSPEN guidelines. Given that each topic area had generated multiple research gaps, we chose to use a ranking system to help readers understand the ones the guideline authors felt were most pressing. We chose to list the top six overall priorities here, as these were the only ones that received 10 or more votes and were clear separated from the others as frontrunners. For the remainder, there were multiple topics (74) that received similar, non-unique, numbers of votes, meaning it is difficult to genuinely say that any one of these priorities is particularly more important than the other. An additional potential concern is that the priorities identified may reflect the authors own research interests, rather than specific needs, though the fact that a broad range of priorities across all areas have been identified, including the top six, suggests this was ultimately not an issue.

The list of research priorities presented here suggests there is still much to do. However, much progress has been made in the past few decades and pediatric PN is now safe, effective, and a vital tool in the support of children who cannot be fed enterally. Future research seems to need to focus on optimizing the composition and administration of PN such that it confers maximal benefit. By listing and highlighting research priorities in this area, this paper acts as a focus point to galvanize the pediatric nutrition research community towards addressing these issues on a grand scale. By making substantial progress in these areas over the next years, we believe that we may promote the future health and well-being of preterm infants, neonates, or older children in need of PN.

## Supplementary information

Supplementary file 1
