# Supplementary material for: Research priorities in pediatric parenteral nutrition: a consensus and perspective from ESPGHAN/ESPEN/ESPR/CSPEN
Source: Pediatr Res. 2021 Sep 2;92(1):61–70. doi: 10.1038/s41390-021-01670-9 (PMC9411056; doi:10.1038/s41390-021-01670-9)
Supplement: Supplementary file 1 [file 41390_2021_1670_MOESM1_ESM.docx]

**Supplementary file 1:** Suggestions of all research questions/priorities collected from the working groups

Energy

- Inadequate basis for any firm recommendations on energy intakes during PN. There is also a potential danger in estimating PN energy needs based on estimated enteral requirements.
- In addition, there has been little work that determines the optimal protein: energy ratio. What is the optimal Protein: Energy ratio for preterm infants?
- The choice of outcome measures deserves to be highlighted. When considering total energy intake, the potential adverse effects of rapid catch-up growth on later metabolic function must be balanced against potential neuro-cognitive benefit. More recently, data has emerged to show that inadequate energy intake is independently associated with the development of severe retinopathy of prematurity (ROP).
- The optimal energy intake in the different phases of critical illness (acute, stable and recovery) of the critical ill child, including the optimal route and doses of macronutrient supplementation, and especially the timing of the parenteral macronutrient supplementation is unknown. Especially in the acute phase one has to account for the endogenous energy production. Larger clinical trials with uniform methodology are needed to further examine the effect of energy intake on protein balance, lean body mass, and clinical outcomes in children on mechanical ventilation.
- What are energy goals during the different phases of critical illness of the critically ill child if PN will be withhold for 1 week?
- Further data for energy intake in children with traumatic brain injury, septic shock, burns, severely undernourished in the different phases of disease and with special focus on the recovery phase.
- Further exploration of the possibilities to measure resting energy expenditure with indirect calorimetry

### Amino Acids

- Optimal glucose and lipid intakes that maximize protein accretion and growth in preterm infants should be determined at various parenteral amino acid intakes in preterm infants.
- Effect of early amino acid administration on growth and development in preterm infants.
- RCT are required to look at the impact on growth of different amino acid intakes during PN in preterm infants.
- Further study on the issue of arginine supplementation in necrotizing enterocolitis (NEC) prevention is clearly needed. In critically ill term infants and older children there are insufficient data are available to draw any firm conclusions on the advisable lower and upper limits for protein intake. The pathophysiological mechanism of the harmful effect of administering proteins in the acute phase of critically illness (eg impaired autophagy) has to be determined.
- The optimal dose and composition of an optimal amino acid mixture for critically ill children remains unclear and has to be further investigated

### Lipids

- More information regarding the long term detrimental effects of ILEs is needed, since aortic stiffness and myocardial function in young adulthood have been associated with the exposure to SO ILEs during neonatal life, but more data are needed to elucidate this further.
- Additional high quality studies are necessary to determine the optimal and/or maximal dose of lipid infusion and the long-term effects on morbidity, growth, and neurodevelopment.
- Whether or not changes in FA profiles are beneficial for the short term and the long term requires further careful evaluation.
- Lipid emulsion containing both DHA and AA at significant amount may provide health benefits to preterm infants and should developed and tested
- Current available evidence suggests that the timing of commencement and dosage of PN lipid in critically ill infants and children may be important, but more research is need to determine in more detail the potential effects on outcomes in relation to the timing of introducing parenteral lipid supply in different pediatric populations
- Current data about lipid clearance during sepsis is conflicting. More studies are needed to define an upper limit of lipid intake in such situations
- More research is needed regarding the effects of the use of composite LEs, including those containing FO, on neonatal morbidity
- High quality RCTs are needed to ascertain the effect of the type and dose of different ILEs on the reversal of PNALD.
- Large randomized studies in critically ill children with acute respiratory are needed to evaluate the effect of limiting pure SO ILE intake or using composite ILE. Similar work is needed regarding the impact of n-3 PUFAs and ILEs in conditions associated with pulmonary hypertension.
- In the context of PNALD, more research is needed into the effect of providing composite ILE with FO on improving clinical outcomes. In relation to this, more work is needed to understand the impact of different ILEs on both the development of, and potential reversal of PN related cholestasis.

### Carbohydrates

- The relationship between excessive glucose intake and dyslipidemia, leading to raised triglycerides and VLDL cholesterol, is not well characterized in critically ill neonates and children, and requires further study
- There is a paucity of research regarding the rate of endogenous glucose production in infants and children, and this data would help guide future recommendation for glucose provision in these groups
- Whilst the PEPaNIC study has demonstrated the benefits of limiting PN([3](#_ENREF_3)), and in turn glucose intakes in critically ill infants and children, more data is needed regarding preterm and term neonates.
- Increased glucose intake and avoiding hyperglycemia might be beneficial and is an area of further research
- The consequences of hypoglycemia on long term outcome in critically ill infants and children has to be investigated
- Hyperglycemia is common especially in sick ELBW infants. How should hyperglycemia be defined in the preterm population, and is it best managed with a reduction in carbohydrate or higher carbohydrate in combination with insulin? Why do some preterm infants tolerate intravenous glucose better than others?

### Fluid and electrolytes

- To identify relationships among fluids and electrolytes received in utero, antenatal and birth hydration status and newborn weight loss during the transition phase.
- To recognize body fluid distribution in transition phase.
- To study enteral and parenteral fluid and electrolyte intake in order to establish optimal requirements including recommendations on electrolyte to macronutrient ratios during the initial postnatal period.
- To differentiate whether a decreased loss of body weight in the first days of life is the result of a delayed postnatal adaptation or an optimal energy balance.
- To determine an “optimal” course of weight loss and gain after birth for neonates with varying gestational age and birth weight.
- To establish whether a safe biochemical control may be assured in neonates in prospective, randomized-control trials comparing standardized versus individualized PN.
- To establish the appropriate composition and safety duration for maintenance parenteral fluid for infants aged 0-3 months.
- Large RCT’s to study the effects of fluid therapy with sodium and chloride concentrations similar to that of plasma, both in preterm infants, neonates and children beyond the neonatal period.
- To study the clinical effects of acidosis.
- To find reliable, low-invasive indicators of hydration status in the premature infant easily applicable in clinical routine.

### Trace elements

- Identify how minimal enteral nutrition in infants and children on long-term PN may sufficiently meet needs for trace elements such as copper, chromium, manganese, molybdenum and selenium.
- Assess safety of available compounds in different populations (very preterm infants, term infants with gastrointestinal failure or infants with PN associated liver disease, older children, etc.).
- Assess compatibility / stability of available compounds in various PN solutions.
- Evaluate trace element contamination in PN products, e.g. manganese and chromium.
- Determine possible benefits of fluoride supplementation of PN.
- Define normal ranges of trace element concentrations in plasma at different age groups and different stress status – if not appropriately established to date and studies aiming to find useful biomarkers for assessment of trace element status.

### Ca and P

- There is a need to develop bedside tools to individually monitor bone mineral (microcrystalline apatite) accretion and bone mineral status ((Ca+P)/body weight)
- The minimum bone mineral accretion to achieve within the first 2-4 weeks of life is currently unknown
- The optimum plasma phosphate concentration is unknown
- There is a need to explore the optimum surrogate parameter for monitoring bone mineral accretion.
- Since the only approved approach for optimizing bone mineral accretion is currently to provide a surplus of Ca and P simultaneously, there is a risk of nephrocalcinosis. This risk, together with prevention strategies for nephrocalcinosis, is not well researched.
- There is insufficient data to enable calculation of phosphate requirements based on calcium deposition and protein accretion in the same way as can be done for adults.
- An absolute level for severe hypophosphatemia is not defined for VLBW infants.
- Further prospective, randomized trials are warranted with focus on the first week metabolic changes that occur on refeeding and should include target values to stabilize electrolytes balances and improve bone mineralization considering nutritional intake and gestational age of the infant, in particular phosphate and potassium.

### Vitamins

- More research is needed in what constitutes the optimal doses and infusion conditions for vitamins in infants and children. Specifically:
- Fat Soluble Vitamins

Vitamin A

- - What constitutes an adequate supply of vitamin A for premature neonates remains controversial and the “adequate” concentration of plasma vitamin A in very low birth weight infants is not known.
  - The benefits, in terms of vitamin A status, safety and acceptability of delivering vitamin A in an intravenous emulsion compared with repeated intramuscular injection should be assessed in a further trial.

Vitamin D

- - More research and high-quality evidence is needed regarding the health effects of additional supplementation with vitamin D (in addition to its role in calcium and phosphate metabolism and bone health), such as prevention of immune-related and infectious diseases, cardiovascular disease, and cancer.
  - The optimum vitamin D requirements of preterm infants on PN are not known.
  - Optimal per oral vitamin D prophylaxis in children receiving parenteral vitamin D should be established.
  - A well-defined threshold for vitamin D acute toxicity has not been established.

Vitamin E

- - There is limited data on how vitamin E requirements change with age, and in particular the upper limit for the dose of vitamin E needs to be established with further, well designed studies.
  - The mechanism involved in the increased risk of infection and hemorrhage in relation to high serum tocopherol levels in preterm infants is unknown and requires further research.
  - Optimal upper level of recommended doses of vitamin E in PN need to be established.
  - Research is needed regarding the use of individualized PN therapy based on an infant's vitamin E status, clinical situation and the type of fat emulsion being used.

Vitamin K

- - Optimal dose of parenteral vitamin K should be established in infants receiving peroral vitamin K supplementation and in infants whose mothers have taken medications that interfere with vitamin K metabolism.
  - Further research is needed to establish optimal biomarker of vitamin K status.
- Water soluble vitamins
  - Current recommendations are expert opinions based on observed biochemical responses to variations in parenteral intake and on comparison with enteral recommendations.
  - Controlled randomized trials in this field are lacking, thus it is recommended to maintain dosages that have been recommended previously and which have been used without apparent harmful effects in clinical practice.

Vitamin C

- - Requirements for vitamin C for preterm infants, term infants and older children are not known.
  - There is no clear clinical indicator for mild and moderate vitamin C deficiency.

Thiamine, Riboflavin, Pyridoxine, Cobalamin, Niacin, Pantothenic Acid, Biotin

- - The precise requirement of these vitamins for parenterally fed infants and children has not yet been defined, and more research and evidence is needed.

Folic Acid

- - The role of folic acid in the establishment of an individual's DNA methylation profile during development is not yet known, nor its involvement in methylation profiles during the life course and, ultimately, the consequences of these profiles for long term health and wellbeing.
  - Additional folic acid supplementation in preterm infants over and above current recommendations is controversial and the literature in this area is limited. More studies are required to ascertain if this is beneficial.

### Venous access

- What are the best landmarks for safe CVC tip positioning in newborns and small children, and does ultrasound guidance prevent complications?
- Does ethanol line lock increase CVC thrombosis and the need for catheter repair, and what is the most cost-effective use of line locks in clinical practice?
- Does heparin or other anticoagulant agent prevent CVC occlusion or thrombosis? What dose, and what mode of administration (flush, infusion, subcutaneous) is most effective?
- Is the use of chlorhexidine as an antiseptic agent safe in young infants, and if not, what is the best replacement?
- What is the most reliable dressing method for short term catheters, and for a tunneled CVC?
- Should children on long-term PN with well healed tunneled CVC be allowed to swim, and what methods should be used to minimize the risk of infection?

### Organizational aspects

- What are the essential elements of a PN ordering process aimed at minimizing the risk of errors?
- What is the basic monitoring protocol essential to ensure safety of patients receiving long-term PN, and what follow up and monitoring should there be for patients who have established enteral autonomy (particularly those with short bowel)?. A minimal data set would be useful.
- How to provide effective light protection to PN fluids in order to prevent generation of oxidants; whether this is relevant to older children on long term PN, as well as the newborn
- RCTs are needed to evaluate the choice of enteral feed whilst on PN, comparing tolerance/efficacy of elemental with polymeric formula and extensively hydrolyzed formula.
- The benefits of cycling PN, and the optimal regimens for doing this in different patient groups, require further research
- Controlled clinical studies to evaluate continuous and intermittent/bolus feeding with respect to energy and nutrient balance are needed.
- A non-invasive test for SIBO remains a priority

### Home PN

- There is a need to develop a network for performing multicenter clinical trials of home PN
- More cost-benefit studies are needed
- A better understanding of the impact of home PN on quality of life of each of the parents, affected child and siblings would be helpful
- Development of pediatric standard formulas for children on PN at home, particularly those that could be kept at room temperature, is a priority
- Research into optimal PN weaning strategies in long-term patients at home is needed
- More long-term outcome data, perhaps as part of an international database or research collaboration, is needed
- There have been reports of older children with certain congenital and/or genetic conditions reducing and even weaning off PN([59](#_ENREF_59)). Multi-centre registry needs to be kept to gain information on the disease course in the largest possible number of children with conditions such as tufting enteropathy, TTC7A deficiency, tricho-hepato-enteric syndrome).
- National or international audits of new treatments for weaning PN e.g. use of GLP-2 in long-term patients at home would help drive practice and the research agenda
- Research into how to prevent central venous catheter (CVC) infection in home PN patients, such as taurolidine locks of other agents would be useful
- Strategies to prevent CVC related thrombosis in home PN patients, such as long-term anti-coagulation treatment, requires new studies
- There is a need to better understand abnormalities of growth, the role of vitamin D, bone density and body composition in the context of home PN
- Better strategies to prevention IFALD and other hepatobiliary disease are needed, and so require more research
- More research into alternatives for venous access in long term PN patients, such as AV shunt, is needed in this population

### Ready to use and standardization

- Is it possible for each centre to manufacture a composition of PN with the current new recommendations or should we aim for a standardized European PN prescription:
- Better data regarding the impact of standardized PN compared to individually tailored PN, including costs and outcomes
- Development and formal trial assessment of multi-chamber PN bags for neonatal and pediatric patients
- Enhanced informatics systems for the prescription, administration and assessment of PN

### Complications

- A definitive diagnosis of a CVC related infection in children remains challenging. Research into newer methods of confirm catheter related blood stream infection (CRBSI) is needed. Differential time to positivity (DTP) of paired peripheral venous and CVC blood cultures may have some potential in the diagnosis of catheter related infections in this setting, but more data is needed
- There are currently no convincing data to support specific recommendations for the duration of therapy for CRBSI, and the optimal duration of therapy for treating CRBSI in children with or without catheter removal has not been established. More work is need in this area
- More trials are needed to establish the role of antibiotic, antifungal and ethanol locks in CVCs as an adjunct to systemic therapy or as an alternative to line removal. Data in these areas is currently lacking.
- Paediatric-specific prospective trials of alteplase for catheter occlusion are needed to establish the optimum treatment for CVC occlusion
- The management of radiographically detected asymptomatic CVC-related thrombosis currently lack evidence, so more research is need in this area
- Chemical or surgical interventions for treating occluded CVCs have not been studied
- More drug and PN brand specific data are needed regarding the impact of medications on PN stability. Currently these data are limited to short reports and product specific. This also applies to interactions with specific equipment and tubing and will vary depending on concentrations and flow rates.
- More trials are need looking at the use of bisphosphonate treatment to improve bone mineral density in infants (some utility has been shown in adults on PN)
- Research is needed regarding the pathogenesis of PN associated liver disease, as currently this is not completely understood
- More definitive, high quality populations specific trials are needed regarding the use of the evidence for the use of ursodeoxycholic acid for the prevention of PN related cholestasis, including data on liver disease and long term outcomes
